# Molecular and Direct Detection Tests for *Treponema pallidum* Subspecies *pallidum*: A Review of the Literature, 1964–2017

**DOI:** 10.1093/cid/ciaa176

**Published:** 2020-06-24

**Authors:** Elitza S Theel, Samantha S Katz, Allan Pillay

**Affiliations:** 1 Division of Clinical Microbiology, Department of Laboratory Medicine and Pathology, Mayo Clinic, Rochester, Minnesota, USA; 2 Division of STD Prevention, Centers for Disease Control and Prevention, Atlanta, Georgia, USA

**Keywords:** *Treponema pallidum*, dark-field microscopy, nucleic acid amplification testing, immunohistochemistry, strain typing

## Abstract

Direct detection methods for *Treponema pallidum* include dark-field microscopy (DFM), direct fluorescence antibody (DFA) testing, immunohistochemistry (IHC), and nucleic acid amplification tests (NAATs). Here, we reviewed the relevant syphilis diagnostic literature to address 2 main questions with respect to *T. pallidum* direct detection techniques: “What are the performance characteristics for each direct detection test for *T. pallidum* and what are the optimal specimen types for each test?” and “What options are available for *T. pallidum* molecular epidemiology?” To answer these questions, we searched 5 electronic databases (OVID Medline, OVID Embase, CINAHL, Cochrane Library, and Scopus) from 1964 to 2017 using relevant search terms and identified 1928 articles, of which 37 met our inclusion criteria. DFM and DFA sensitivities ranged from 73% to 100% in cases of primary syphilis; and while sensitivity using silver stain histopathology for *T. pallidum* was generally low (0%–41%), higher performance characteristics were observed for *T. pallidum*–specific IHC (49–92%). Different genes have been targeted by *T. pallidum*–specific NAATs, with the majority of studies indicating that sensitivity is primarily dependent on the type of collected biological sample, with highest sensitivity observed in primary lesion exudate (75–95%). Given the rising incidence of syphilis, the development of direct, Food and Drug Administration–cleared *T. pallidum* NAATs should be considered an immediate priority.

The number of primary and secondary cases of syphilis caused by infection with *Treponema pallidum* subspecies *pallidum* (herein *T. pallidum*) has steadily increased in the United States since the early 2000s. This has resulted in a nearly 73% increase in the overall number of cases reported to the Centers for Disease Control and Prevention (CDC) in 2017 as compared to 2013, and is apparent across sexual behaviors, ethnicities, and gender, leading to a more than 40% increase in congenital syphilis cases over the past year [1]. In the absence of treatment, syphilis can progress through multiple stages and present with a variety of different clinical manifestations, collectively making laboratory testing, alongside a careful clinical evaluation, invaluable for establishing the diagnosis. Diagnostic testing for syphilis can be challenging, however, as the organism cannot be grown in routine culture and there is no single assay with sufficient sensitivity and specificity to identify all stages of disease. Currently, diagnosis remains primarily dependent on serologic evaluation for antibodies to *T. pallidum* using both treponemal and nontreponemal assays, via either a traditional or reverse algorithmic approach [2]. Although serologic testing is associated with high sensitivity (>95%) during secondary and later stages of disease, the ability of these assays to detect cases of primary infection is significantly diminished, with studies suggesting that 14% to 46% of patients with primary syphilis may be seronegative depending on the method used [2–5]. Additional limitations of serologic testing include lifelong seropositivity using treponemal assays and the possibility of serofast status, positive results by nontreponemal assays in successfully treated patients, further complicating the interpretation of results.

In an effort to mitigate these limitations, different direct detection methods for *T. pallidum* have been developed, including direct visualization of spirochetes from lesion exudate by dark-field microscopy (DFM) or direct fluorescence antibody (DFA) testing, histopathology using silver stains or immunohistochemistry (IHC), and nucleic acid amplification tests (NAATs). Notably, no direct detection assay has been cleared by the Food and Drug Administration (FDA) for marketing in the United States. Given the limited sensitivity of current serologic assays during primary disease, direct detection assays may be most beneficial in the early stage(s) of infection, yet they are not widely available or performed at local, reference, or public health laboratories (PHLs). There are numerous reasons for this, including challenges associated with accurate assay interpretation and maintaining technological expertise, limited specimen stability or availability for assay validation, and reagent accessibility. However, with the continued increase in syphilis rates, including primary syphilis, hospitals and laboratories may need to re-evaluate the need for direct detection methods, particularly in areas of the United States with a high incidence of disease. This literature review provides an updated summary of the utility and performance characteristics of direct detection techniques for *T. pallidum*.

## METHODS

### Strategy

**Figure 1.**
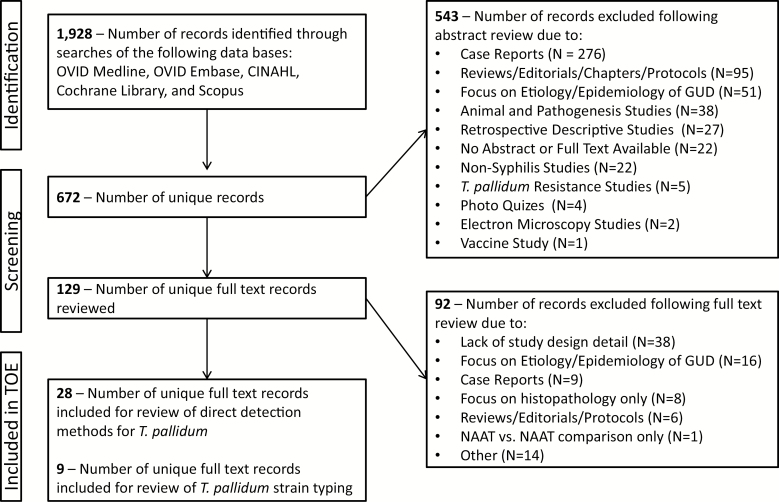
Summary of literature review process to identify relevant studies to answer the 2 key questions related to direct detection of *T*reponema *pallidum*. The term “Other” includes excluded articles due to focus on animal and pathogenesis studies (n = 4), blood donor screening (n = 3), electron microscopy (n = 2), lack of direct detection methods (n = 2), non-syphilis studies (n = 2), and *T. pallidum* resistance studies (n = 1). Abbreviations: GUD, genital ulcer disease; NAAT, nucleic acid amplification test; TOE, tables of evidence.

The APHL identified clinical, public health, and laboratory subject matter experts on *T. pallidum* who were tasked to identify “key questions” in the field of diagnostic testing for syphilis. Among the identified topics, 2 were focused on direct detection methods for *T. pallidum* and specifically included the questions: “What are the performance characteristics for each direct detection test for *T. pallidum* and what are the optimal specimen types for each test?” and “What options are available for *T. pallidum* molecular epidemiology?” To address these questions, a literature review of papers published between January 1964 and June 2017 was conducted by searching OVID Medline, OVID Embase, Cumulated Index to Nursing and Allied Health Literature (CINAHL), the Cochrane Library, and Scopus with a combination of agreed-upon “keywords” as listed in Supplementary Table 1. The search strategy was executed by expert librarians at the CDC and initial results were screened to remove duplicates. All unique titles and abstracts (as available) were assessed by the authors for relevance to the key questions, and the full text for all applicable articles was retrieved (as available) and reviewed. Inclusion criteria for this review included primary literature assessing the performance characteristics of direct detection techniques for *T. pallidum*, including DFM, DFA, IHC, NAATs, and molecular strain typing studies. Article exclusion criteria for this literature review included the following: nonhuman studies, surveillance- or epidemiology-focused studies, editorials, photo quizzes, basic research studies, studies without direct detection or molecular methods, non-English articles, articles without available abstracts, conference abstracts, procedures, nonprimary literature, studies focused on non–*T. pallidum* subsp. *pallidum*, and case reports. A summary of the literature review is presented in Figure 1.

### Data Extraction

Each of the full-text manuscripts included in this review was evaluated in detail and the data recorded in a “table of evidence” (TOE). A TOE was completed for each key question, which included the assessment and summary of study data for each of the following topics: (1) description of the study type, design, population, and setting; (2) reported findings and quantitative results related to direct detection methods for *T. pallidum*; (3) overall quality including strengths/weaknesses or limitations; and (4) relevance to the topic of direct *T. pallidum* detection methods and/or overall importance (Supplementary Tables 2 and 3). These TOEs were presented at the APHL Laboratory Diagnosis of Syphilis meeting in Atlanta, Georgia, on 28–29 November 2017 to a group of more than 50 clinical, laboratory, and PHL experts on syphilis. Preliminary answers to the key questions were identified and revised based on the TOE, and areas of additional research were identified. A summary of these findings and discussion is presented here.

## RESULTS AND DISCUSSION

### Study Selection

A total of 1928 articles were identified, among which 672 were unique. Of those, 543 were excluded due to the aforementioned exclusion criteria (n = 521) or lack of accessibility to the abstract or full text (n = 22). The remaining 129 full-text articles were reviewed in detail, of which 92 were excluded as a result of lack of study design detail (n = 38), use of direct detection techniques to establish etiology or epidemiology of genital ulcer disease (GUD) (n = 16), case reports (n = 9), focus on histopathology only (n = 8), reviews, editorials or protocols (n = 6), or due to a focus on other topics not applicable to the key questions (n = 14) (Figure 1). In total, 37 articles met the strict inclusion criteria, including 28 studies evaluating the performance characteristics of direct detection techniques for *T. pallidum* and 9 manuscripts discussing *T. pallidum* molecular strain typing.

### What Are the Performance Characteristics for Each Direct Detection Test for *Treponema pallidum* and What Are the Optimal Specimen Types for Each Test?

Classically, the reference method for direct detection of *T. pallidum* has been the rabbit infectivity test (RIT), which involves inoculation of patient specimens into a rabbit testis and, if positive, results in seroconversion and/or identification of spirochetes by DFM from lesions or by histopathology [6]. Although originally considered to be a sensitive method for detection of *T. pallidum,* with a limit of detection of 1–3 spirochetes and a 50% infectious dose of 23 spirochetes, more recent studies suggest that sensitivity of the RIT is low (<50%) in patients with primary syphilis or neuroinvasive disease [6-9]. Additionally, due to the challenges associated with the RIT, including access to and maintenance of rabbit colonies, prolonged incubation times (2–6 months), and expense, this method is no longer applicable in the clinical setting, although it continues to be utilized for isolation and cultivation of *T. pallidum* strains in specialized laboratories for nondiagnostic, research purposes. As a result, alternative direct detection techniques, including DFM, DFA, silver staining, IHC, and NAATs, have been developed and evaluated for clinical utility.

#### Dark-field Microscopy

Dark-field microscopy is used at the point-of-care due to the strict specimen stability requirements [10]. The method is typically performed on moist primary or secondary lesions, by directly applying or transferring serous exudate (free of red blood cells) onto a sterile microscope slide. Dark-field microscopy is not recommended to be performed on oral lesions due to the presence of *Treponema denticola*, which is present as normal flora in the oral cavity of most individuals and is indistinguishable morphologically from *T. pallidum* [5]. Within 20 minutes of collection, the sample should be examined under a light microscope equipped with a double or single reflecting dark-field condenser for the presence of spirochetes meeting both morphologic and motility criteria characteristic of *T. pallidum* [10]. Accurate results by DFM are dependent on rapid access, ideally within 20 minutes, to a dark-field microscope (to minimize loss of motility) and on technologist proficiency and expertise in identifying *T. pallidum* and differentiating it from other spirochetes that may be normal flora (eg, *Treponema refringens* in genital regions). Among patients with primary syphilis, defined by clinical presentation and laboratory findings (eg, serology and/or NAAT), sensitivity and specificity of DFM ranged from 75% to 100% and 94% to 100%, respectively (Table 1) [11–15]. In secondary syphilitic lesions, similarly classified based on clinical history alongside serologic studies and/or NAAT, DFM sensitivity ranged from 58% to 71%; and, although reported by a single study, specificity was high at 100% (Table 1) [13, 15, 16]. Dark-field microscopy has also been evaluated as a method for rapid diagnosis of congenital syphilis by direct examination of amniotic fluid (AF). Compared with RIT, DFM sensitivity in AF ranged from 42% to 86% across the included studies, with excellent specificity (100%) (Table 2) [21, 22]. Reports evaluating DFM performance in cerebrospinal fluid (CSF) or other body fluids, other than AF, did not meet the inclusion criteria for this review. Despite relatively good performance characteristics, the requirement for microscopes with dark-field capabilities and technologist expertise, both of which need to be in close and immediate proximity to patient examination rooms, most clinics and hospitals are unlikely to have the capacity to routinely perform DFM. However, given the rising incidence of primary and secondary syphilis, DFM may be an important diagnostic assay to maintain in sexually transmitted disease clinics and PHLs located in high-incidence regions, with the caveat that clinic and laboratory staff must be able to evaluate specimens within 20 minutes of collection.

**Table 1.** Summary of the Performance Characteristics for Direct Detection of *Treponema pallidum* in Adults Using Dark-field Microscopy, Direct Fluorescence Antibody Testing, Silver Staining, or Immunohistochemistry

Method	Specimen Source	Collection Method	Assay Target	Syphilis Stage	Sensitivity, %	Specificity, %	Comparator Method	References
Dark-field microscopy	Lesion exudate (not oral)	Direct or loop application of exudate onto slide	Live *T. pallidum* spirochete	Primary	75–100	94–100	Clinical presentation and laboratory testing (eg, serology, NAAT)	[11–16]
				Secondary	58–71	100		
Direct fluorescence antibody	Primary or secondary lesion exudate (not oral)	Direct or loop application of exudate onto slide	47–48 kDa *T. pallidum* protein (H9–1 mAb)	Primary	73–100	100	DFM	[11, 12]
			pAbs	Primary	84–87	91–100		[14]
Silver stain^a^	FFPE tissue biopsy	Biopsy	*T. pallidum* spirochete	Secondary	0–41	N/A	DFM or clinical presentation and laboratory testing (eg, serology, NAAT)	[17–19]
Immunohistochemistry	Primary or secondary lesion biopsy (FFPE)	Biopsy	pAb to *T. pallidum* via ABC technique	Secondary	49–92	100		[13, 16–18]

Abbreviations: ABC technique, avidin-biotin peroxidase complex technique; DFM, dark-field microscopy; FFPE, formalin-fixed, paraffin-embedded; mAb, monoclonal antibody; N/A, not applicable; NAAT, nucleic acid amplification test; pAb, polyclonal antibody.

^a^Includes the Steiner, Dieterle, and Warthin-Starry stains

**Table 2.** Summary of the Performance Characteristics for Direct Detection of *Treponema pallidum* in Congenital Syphilis Compared With Clinical Presentation, Laboratory Findings, and Rabbit Infectivity Testing

Method	Specimen Source	Assay Target	Sensitivity, %	Specificity, %	Comparator Method	References
Dark-field microscopy	Amniotic fluid	*T. pallidum spirochete*	42–86	100	RIT	[21, 22]
Silver stain (Dieterle)	FFPE autopsy tissue sections (various organs)	*T. pallidum* spirochete	37–41	NR	Indirect IFA	[23]
NAAT	Amniotic fluid	*tpp47*	75–100	100	RIT	[21, 22, 24]
	Neonatal cerebrospinal fluid	*tpp47*	60–75	97–100		[22, 25, 26]
	Neonatal whole Blood or serum	*tpp47*	67–94	90–100		[22, 25]

Abbreviations: FFPE, formalin-fixed, paraffin-embedded; IFA, immunofluorescence assay; NAAT, nucleic acid amplification test; NR, not reported; RIT, rabbit infectivity test.

#### Direct Fluorescence Antibody

Direct fluorescence antibody testing for *T. pallidum* can be performed as an alternative to DFM on serous fluid collected from syphilitic lesions, and on other body fluids (eg, CSF, AF, etc). This method involves application and ethanol fixation of the specimen to a microscope slide, followed by staining with either fluorescently conjugated monoclonal or polyclonal antibodies to *T. pallidum* and examination for the presence of fluorescent spirochetes. Although such antibodies may be available commercially, none are currently FDA cleared for use in clinical laboratories. A key advantage of DFA over DFM includes the ability to submit air-dried or acetone-fixed samples to the laboratory for examination, circumventing the DFM requirement of immediate evaluation. Additionally, although technologist expertise is still necessary, interpretation of DFA is significantly more straightforward (ie, presence or absence of sufficient fluorescence) than DFM, which depends on accurate assessment of spirochete morphology and motility consistent with *T. pallidum.* Among the included studies in this review, 2 evaluated performance of the H9–1 monoclonal antibody (mAb) to *T. pallidum* in primary and secondary lesions, with a sensitivity ranging from 73% to 100% and specificity of 100% compared with DFM (Table 1) [11, 12]. The H9–1 mAb is specific to *T. pallidum* and *T. pallidum* subsp. *pertenue* (*T. pertenue*), detecting a 47 to 48 kDa protein from *T. pallidum*, and does not react with other commensal spirochetes [12]. Therefore, DFA using this antibody may be performed on oral or anogenital specimens with limited risk of false-positive results. A single study evaluating polyclonal antibodies for DFA on syphilitic lesions was included in this review, which showed high sensitivity and specificity, 84% to 87% and 91% to 100%, respectively, compared with either DFM alone or with a clinical diagnosis based on history, DFM, and syphilis serology results among 350 patients [12]. Overall, despite the similar sensitivity observed between polyclonal and monoclonal antibodies for DFA, use of the H9–1 mAb is preferred due to higher associated specificity as compared with polyclonal antibodies. However, these studies were all performed decades ago, with no recent publications evaluating DFA, underscoring the limited accessibility of reagents and utility of this method in current clinical practice.

#### Silver Staining and Immunohistochemistry

Multiple different silver stains, including Steiner, Warthin-Starry, and Dieterle stains, have been evaluated for the detection of *T. pallidum* in formalin-fixed, paraffin-embedded (FFPE) tissue biopsies, principally from primary or secondary syphilis lesion biopsies. Compared with either DFM performed on exudate material from these lesions or with clinical diagnosis and staging based on presentation and serologic results, the sensitivity of silver staining of FFPE tissue biopsies ranged from 0% to 41% (Table 1) [17–20]. Although specificity was not directly discussed, the majority of publications described the various challenges associated with interpretation, including staining of melanin and reticulin fibers, which can mimic the appearance of spirochetes in tissue. Silver staining has also been evaluated in cases of fetal demise resulting from congenital syphilis, and sensitivity across organ sections collected at autopsy ranged from 37% to 41% compared with an indirect immunofluorescence assay (Table 2) [23]. Generally, due to challenges associated with stain interpretation and limited sensitivity alongside availability of alternative diagnostic techniques (both direct and indirect), silver staining is not routinely used for the diagnosis of syphilis.

Numerous different IHC methods for detection of *T. pallidum* in FFPE tissue sections exist; however, the avidin-biotin peroxidase complex (ABC) technique has most frequently been evaluated across studies. Briefly, this method involves heat-induced *T. pallidum* epitope exposure in tissue sections, followed by incubation with polyclonal rabbit anti–*T. pallidum* immunoglobulin G (IgG) antibodies. Subsequently, biotinylated antibodies to rabbit IgG are added, followed by a final incubation with peroxidase-conjugated avidin-biotin complex and visualization of treponemes. Compared with a clinical diagnosis of secondary syphilis, the ABC IHC method shows excellent specificity across studies, with a sensitivity ranging from 49% to 92% (Table 1) [13, 16–18]. Many of these studies also evaluated alternative direct detection methods on secondary syphilitic lesions and concluded that the ABC technique for staining *T. pallidum* spirochetes in tissue was significantly more sensitive and specific than either DFM on lesion exudate or silver staining of FFPE tissue biopsies.

#### Nucleic Acid Amplification Testing

Nucleic acid amplification tests have been developed and evaluated for the detection of *T. pallidum* DNA from various specimen sources and disease stages, yet none are commercially available to date. These molecular assays have differed in method (eg, classic polymerase chain reaction [PCR], nested PCR, quantitative PCR, reverse transcriptase PCR), design (singleplex vs multiplex assays with targets for alternative causes of GUD) and *T. pallidum* gene target. Numerous *T. pallidum* genes have been evaluated for molecular diagnostic purposes, including those encoding treponemal surface or subsurface lipoproteins (ie, treponemal membrane protein A [*tmpA*], subsurface lipoprotein 4D [*4D*], basic membrane protein [*bmp*] and *T. pallidum* 47 kDa lipoprotein [*tpp47*]) and the DNA polymerase I gene (ie, *polA*) [15, 27–30]. Among these targets, NAATs based on the detection of *tpp47* and *polA* have been described and evaluated most frequently and are the focus in this review. Generally, NAATs using these genes show excellent specificity across studies, ranging from 97% to 100%, whereas sensitivity is largely dependent on the specimen source tested rather than on the *T. pallidum* gene targeted (Table 3). It is important also to remain cognizant of the different comparator reference methods (e.g., DFM vs clinical presentation alongside serologic results used to define primary syphilis) and national guidelines (e.g., CDC vs Canadian vs European guidelines) used to stage patients or define disease across these studies.

**Table 3.** Summary of the Performance Characteristics for Nucleic Acid Amplification Tests for *Treponema pallidum* in Adults

			Sensitivity, NAAT Target, %		Specificity, NAAT Target, %		
Specimen Source	Collection Method	Syphilis Stage	tpp47	polA	tpp47	polA	References
Lesion	Exudate swab	Primary	75–95	72–87	98–100	98–98	[15, 31–37]
		Secondary	20–86	44			
Lesion biopsy	Biopsy	Secondary	26–75^a^	67	100	NR	[16, 17, 19, 20]
CSF	Lumbar puncture	Neurosyphilis	50–77	70	100	100	[35, 38]
Whole blood/serum/plasma	Venipuncture	Primary	12–55	50	100	100	[15, 19, 31, 35, 36, 39, 40]
		Secondary	15–47	44–45	100	100	
		Latent	0–44	0–62	100	NR	

The comparator methods and diagnostic guidelines used to evaluate these NAATs varied between studies. Refer to the text for details.

Abbreviations: CSF, cerebrospinal fluid; NAAT, nucleic acid amplification test; NR, not reported.

^a^75% sensitivity was found using fresh, frozen tissue [16].

The highest overall sensitivity of NAAT for *T. pallidum* detection was observed in primary lesion exudate collected by sterile swab and ranged from 75% to 95% and 72% to 87% for the *tpp47* and *polA* targets, respectively (Table 3) [15, 31–37]. Performance of *tpp47* or *polA* NAATs compared with only DFM in primary lesions showed high positive agreement, ranging from 87% to 95% [32, 33, 35]. Molecular testing of exudate swabs collected from patients classified with secondary syphilis showed a wide range in sensitivity for the *tpp47* target, 20% to 86%, yet limited sensitivity by the *polA* NAAT (43%) (Table 3) [15, 32, 34, 35]. Of note, only a single study assessing *polA* NAAT in secondary syphilis was included in this review, and of the 3 publications assessing *tpp47* performance, 2 reported a sensitivity of 80% or higher [15, 32, 34]. These ranges in sensitivity may be attributable to multiple factors, including assay method, but may also reflect inadequate or inconsistent sampling of the lesion, particularly those requiring scraping to collect exudate material. Collectively, NAATs performed directly from primary or secondary lesion exudate may be used to establish a diagnosis of syphilis, with negligible difference in performance characteristics between the *tpp47* and *polA* targets.

Nucleic acid amplification tests have also, although infrequently, been applied to FFPE tissue biopsies of secondary syphilitic lesions, with only moderate sensitivity of up to 67% reported among studies using either *tpp47* or *polA* as the target (Table 3) [16, 17, 19, 20]. This limited sensitivity may in part be due to the formalin fixation process, resulting in DNA degradation and cross-linkage, ultimately leading to inhibition of DNA amplification [41].

Neurosyphilis remains a challenging diagnosis to establish due to imperfect serologic markers of infection and nonspecific clinical manifestations. Direct detection of *T. pallidum* in CSF using NAATs has been evaluated as a possible additional tool to aid in the diagnosis neuroinvasive disease. Among the largest studies evaluating this utility of *T. pallidum* NAATs on CSF, Castro and colleagues [38, 42] enrolled 124 patients with reactive *T. pallidum* serologic results in serum and, using the 2008 European Guidelines on the Management of Syphilis, determined that 33 patients met the criteria for neurosyphilis, while 91 did not. Among these patients, 25 (76%) and 23 (70%) of the 33 patients with neurosyphilis and 12 (13%) and 7 (8%) of the 91 patients without neurosyphilis were positive by the *tpp47* and *polA* NAATs, respectively (Table 3) [38]. A second study evaluating the *tpp47* NAAT in CSF collected from 6 patients diagnosed with neurosyphilis based on CDC criteria found that only 3 were positive [35]. *Treponema pallidum* NAATs performed on CSF have also been investigated as an additional diagnostic aid for cases of suspected congenital syphilis in neonates. Among the 3 included studies in this review, CSF samples from neonates born to mothers with confirmed and untreated syphilis during pregnancy were tested by the *tpp47* NAAT, which resulted in varied sensitivity, from 60% to 75%, as compared with the RIT performed on the same sample (Table 2) [22, 25, 26]. Overall, these studies suggest that *T. pallidum* NAATs on CSF from symptomatic adults and neonates cannot be used to rule out infection due to its low sensitivity. Their role as adjunct tests for diagnosis of neurosyphilis or congenital syphilis remains undefined.

Whole blood and blood fractions (i.e., serum, plasma, peripheral blood mononuclear cells) have also been evaluated as possible specimen sources for *T. pallidum* NAATs in patients with primary, secondary, or latent syphilis. Irrespective of disease stage, however, molecular assays are insensitive for the detection of *T. pallidum* DNA in any blood fraction, with a sensitivity ranging from 0% to 55% and 0% to 62% across studies for *tpp47* and *polA* NAATs, respectively (Table 3) [15, 19, 31, 35, 39, 40]. Interestingly, capillary blood collected from the earlobe following scraping is an alternative specimen type that has been evaluated for direct detection of *T. pallidum* and is based on the hypothesis that *T. pallidum* may reside in capillary beds during latent infection. Using this sample type, sensitivity of NAATs using *polA* or *tpp47* gene targets ranged from 54% to 66% [40, 43, 44]. *Treponema pallidum* molecular testing of serum or whole blood collected from neonates born to mothers with confirmed, untreated syphilis during pregnancy is associated with significantly higher sensitivity, ranging from 67% to 94%, in comparison to the reference standard RIT method (Table 2) [22, 25]. Collectively, these studies suggest that *T. pallidum* NAATs performed on blood collected from adults presenting with primary, secondary, or latent infection are insensitive and cannot be relied upon to exclude infection. However, testing of neonatal blood or serum may be considered a useful adjunct method for diagnosis of congenital disease.

Amniotic fluid, collected from pregnant mothers with *T. pallidum*, has also been evaluated as an alternative specimen source for diagnosis of congenital syphilis. Molecular testing of AF using *tpp47*-based NAATs has shown high sensitivity (75% to 100%) across 3 studies in comparison to the RIT reference standard (Table 2) [21, 22, 24]. Overall, published literature on *T. pallidum* NAATs as complementary methods for the diagnosis of congenital syphilis indicates that the highest yield samples are AF, neonatal blood, and CSF, and furthermore, evaluation of all 3 sources may provide the highest overall sensitivity for this congenitally acquired infection.

### What Options Are Available for Molecular Epidemiology and What Should Be Considered for Specimen Collection/Preservation?

The ability to differentiate among *T. pallidum* strains might provide invaluable information regarding the epidemiology of this spirochete, which could be used to enhance surveillance and infection control at the local or regional level. Additionally, *T. pallidum* strain typing from clinical specimens can help distinguish cases of relapse from those of reinfection, discriminate among strains associated with certain clinical manifestations (eg, neuroinvasive vs nonneuroinvasive strains), and contribute to a better understanding of the pathogenesis and transmission of this organism. Due to an inability to perform routine in vitro culture for *T. pallidum*, subtyping of *T. pallidum* has relied entirely on a targeted molecular approach using clinical specimens and was first described by Pillay and colleagues in 1998 [45]. Briefly, this method involves interrogation of 2 *T. pallidum* genes, the acidic repeat protein (*arp*) gene and the *T. pallidum* repeat gene subfamily II (*tprE*, *tprG*, and *tprJ*) genes. The *arp* gene contains 60 base pair (bp) repeats, with the number of repeats varying among strains. This repeat region is amplified using NAAT, the number of 60-bp repeats is determined, with 3 to 25 such repeats described to date. In contrast, following amplification of the *tpr* genes, restriction fragment length polymorphism analysis is performed by enzymatic digestion with the *MseI* endonuclease. The resulting combinations of *tpr* gene fragments are analyzed and assigned a letter, “a” through “p.” Using this typing scheme, the reference laboratory strain of *T. pallidum* (Nichols) has been subtyped as 14a, and more than 280 different typed *T. pallidum* strains have now been reported worldwide [45, 46]. This approach is limited, however, in its ability to differentiate among the most commonly circulating strains (ie,14d and 14f) [47]. In an effort to provide further discriminatory power among strains, additional *T. pallidum* genes have been evaluated, including determining the number of G repeats in the *rpsA* gene (range of possible repeats, 8–11) or analysis of an 84-bp region of the *tp0548* gene, among others [47, 48]. Evaluation of these additional genes has allowed for the identification of unique strain types within the higher-level classification scheme using the *arp/tpr* analysis. For example, sequence analysis of the *tp0548* 84-bp region among *T. pallidum* strains with 14 different *arp/tpr* strain types revealed 24 unique subtypes [47]. This additional level of discrimination can lead to improved epidemiologic surveillance of circulating strains and outbreak events in communities and may be of increasing importance as syphilis incidence increases throughout the United States.

Typing studies have been performed on many of the same specimen sources (eg, lesion exudate, CSF, blood, etc), as described for direct detection of *T. pallidum* by NAAT. Specimens are first screened for the presence of *T. pallidum* nucleic acid using well-described gene targets, including *tpp47*, *polA*, and *bmp*, with only screen-positive samples undergoing subsequent subtyping using *arp/tpr* and strain typing using the aforementioned gene analyses. Sensitivity of the initial screening NAATs for typing purposes is primarily dependent on specimen source, as indicated above, with the highest sensitivity reported from lesion exudate (*polA*: 94–97%; *tpp47*: 91–97%; *bmp*: 97%), followed by CSF (*tpp47*: 56%) and blood (*polA*: 19–39%; *tpp47*: 18–41%; *bmp*: 18%) [31, 43, 44, 48–51]. Strain typing requires a higher copy number of *T. pallidum* genomic DNA since some (16–50%) NAAT-positive samples were not able to be fully typed by both *arp* and *tpr*. Successful *arp* analysis of initially *polA* or *tpp47* NAAT-positive samples ranges from 45% in blood to 92% in lesion exudate, whereas *tpr* success rates trend higher, from 85% to 100% in blood and exudate material [31, 49, 51]. Complete *arp* and *tpr T. pallidum* subtyping from screen-positive samples varies among studies and specimen types, ranging from 34% to 93% in blood fractions, 64% to 100% in lesion exudate, 76% to 84% in capillary blood from earlobes, and approximately 46% from CSF [31, 43, 44, 46, 48–51]. Analysis of the *tp0548* gene in initially NAAT-positive samples has been associated with a high success rate in blood, approximately 84%. However, due to lower success rates of *arp* typing, complete strain typing with *arp, tpr*, and *tp0548* is significantly lower, ranging from 40% to 51% [43, 49].

Collectively, strain typing of *T. pallidum* is most frequently successful from lesion exudate following initial screening with an NAAT targeting *polA* or *tpp47*. However, molecular typing is of limited use in routine clinical practice as it does not impact either clinical decisions or treatment selection. Typing of *T. pallidum* strains is primarily useful for epidemiologic purposes to monitor strain variance over time and across geographic regions. Although not specifically addressed in this review, molecular typing studies may have an increasingly influential role in establishing the presence or absence of genetic determinants of azithromycin or doxycycline resistance across *T. pallidum* strains. *Treponema pallidum* remains universally susceptible to penicillin G; however, alternative treatment regimens, including azithromycin or doxycycline, are required for those with significant allergic reactions to penicillin. Resistance to these alternative antibiotics, particularly to macrolides, is increasing across Europe and Asia, which may necessitate more routine molecular evaluation of strains to determine susceptibility profiles prior to treatment [52, 53].

### Research and Development Priorities

Numerous areas for future research and assay development were identified during this review. Perhaps most paramount among these is defining an agreed-upon reference standard against which direct detection assays for *T. pallidum* can be compared. Apparent from this review is the wide variety of comparison assays and/or clinical reference criteria used for assay evaluation; this lack of standardization significantly limits our ability to accurately cross-compare the performance characteristics of different *T. pallidum* direct detection assays. Additional areas for future focus include the collection of improved and more current data associated with specimen collection, particularly for exudate material submitted for NAAT from suspected primary or secondary syphilitic lesions—what is the definition of a “quality” specimen, what swab types or other collection devices should be used, should specimens be submitted dry or in transport media, what transport temperature is recommended, what is the specimen stability, etc? Additionally, although some studies evaluated direct detection techniques in individuals with human immunodeficiency virus (HIV) and those without HIV, data on the performance characteristics of these assays between these and other patient populations (ie, immunocompromised hosts) remain limited. Development of both point-of-care and/or laboratory-based multiplex molecular assays for detection of common causes of GUDs in the United States should become a priority, especially given the rising incidence of syphilis and the already high rate of herpes simplex virus infections throughout the country. Submission of such assays for FDA clearance should be considered a priority by manufacturers and laboratories with the capability of developing and validating direct detection methods for syphilis.

## Supplementary Data

Supplementary materials are available at *Clinical Infectious Diseases* online. Consisting of data provided by the authors to benefit the reader, the posted materials are not copyedited and are the sole responsibility of the authors, so questions or comments should be addressed to the corresponding author.

ciaa176_suppl_Supplemental_Tables_1-3

## Notes

***Supplement sponsorship.*** This article appears as part of the supplement National Coalition of STD Directors, sponsored by the CDC.

***Potential conflicts of interest.*** The authors: No reported conflicts of interest. All authors have submitted the ICMJE Form for Disclosure of Potential Conflicts of Interest.

Presented in part: These findings were presented, in part, at a meeting on laboratory diagnosis of syphilis in Atlanta, Georgia, 28–29 November 2017, facilitated by the Association of Public Health Laboratories (APHL).
