# Supplementary material for: Molecular and Direct Detection Tests for Treponema pallidum Subspecies pallidum: A Review of the Literature, 1964–2017
Source: Clin Infect Dis. 2020 Jun 24;71(Suppl 1):S4–S12. doi: 10.1093/cid/ciaa176 (PMC7312206; doi:10.1093/cid/ciaa176)
Supplement: ciaa176_suppl_Supplemental_Tables_1-3 [file ciaa176_suppl_supplemental_tables_1-3.docx]

**Supplemental Table 1. Summary of Literature Search.** The literature search for this review included the following search terms: (Syphilis OR *Treponema pallidum*) AND (Genital ulcer disease OR primary syphilis OR secondary syphilis OR tertiary syphilis OR congenital syphilis OR ocular syphilis) AND (diagnosis OR lesions OR polymerase chain reaction OR PCR OR nucleic acid amplification test OR NAAT OR multiplex test OR silver stain OR silver staining OR immunohistochemistry OR IHC OR rabbit infectivity testing OR RIT OR direct detection OR dark field microscopy OR darkfield microscopy OR dark-field microscopy OR darkground microscopy OR direct fluorescent antibody OR DFA OR direct fluorescent antibody for *T. pallidum* OR DFA-TP OR direct fluorescent antibody tissue test for *T. pallidum* OR DFAT-TP).

English; 1960-

**Search Strategy:**

| **Database** | **Strategy** | **Run Date** |
| --- | --- | --- |
| **Medline**  **(OVID)**  **1946-** | Treponema pallidum/ip OR Syphilis/di OR (detect* adj4 (Treponema pallidum OR T pallidum OR syphilis)).ti,ab.  AND  *Diagnosis/ OR (lesions OR polymerase chain reaction OR nucleic acid amplification test OR multiplex test* OR silver stain* OR immunohistochemistry OR immunohistochemical stain* OR rabbit infectivity test* OR direct detection OR direct test* OR dark field microscopy OR darkfield microscopy OR darkground microscopy OR dark ground microscopy OR direct fluorescent antibody OR DFAT-TP).ti,ab.  English, 1960- | 7/19/2017 |
| **Embase**  **(OVID)**  **1947-** | *Treponema pallidum/ OR Syphilis/di OR (detect* adj4 (Treponema pallidum OR T pallidum OR syphilis)).ti,ab.  AND  *Diagnosis/ OR (lesions OR polymerase chain reaction OR nucleic acid amplification test OR multiplex test* OR silver stain* OR immunohistochemistry OR immunohistochemical stain* OR rabbit infectivity test* OR direct detection OR direct test* OR dark field microscopy OR darkfield microscopy OR darkground microscopy OR dark ground microscopy OR direct fluorescent antibody OR DFAT-TP).ti,ab.  English; 1960- ; Exclude Medline Journals | 7/19/2017 |
| **CINAHL**  **(Ebsco)**  **1982-** | (MH "Treponema pallidum"/IP) OR (MH Syphilis/DI) OR (TI (detect* N4 (Treponema pallidum OR T pallidum OR syphilis))) OR (AB (detect* N4 (Treponema pallidum OR T pallidum OR syphilis)))  AND  (MH Diagnosis) OR (TI (lesions OR "polymerase chain reaction" OR "nucleic acid amplification test" OR "multiplex test*" OR "silver stain*" OR immunohistochemistry OR "immunohistochemical stain*" OR "rabbit infectivity test*" OR "direct detection" OR "direct test*" OR "dark field microscopy" OR "darkfield microscopy" OR "darkground microscopy" OR "dark ground microscopy" OR "direct fluorescent antibody" OR DFAT-TP)) OR (AB (lesions OR "polymerase chain reaction" OR "nucleic acid amplification test" OR "multiplex test*" OR "silver stain*" OR immunohistochemistry OR "immunohistochemical stain*" OR "rabbit infectivity test*" OR "direct detection" OR "direct test*" OR "dark field microscopy" OR "darkfield microscopy" OR "darkground microscopy" OR "dark ground microscopy" OR "direct fluorescent antibody" OR DFAT-TP))  English, 1960- | 7/19/2017 |

            Key to understanding OVID syntax:

/ = Subject Heading

Exp = explimit of detectione

ADJ? = (adjacent to) within ? number of words

.mp. = title, abstract, keyword heading word, subject heading word

* = truncation character

| **Supplemental Table 2. Table of Evidence summarizing the performance characteristics of direct detection methods for *T. pallidum*.** | | | | | | | | |
| --- | --- | --- | --- | --- | --- | --- | --- | --- |
| **Citation** | **Description of study type, design, population, and setting** | | **Reported findings, quantitative results related to key question** | | **Overall quality; strengths/weaknesses or limitations** | | **Relevance to the key question and/or overall importance** | |
| **1. Dark Field Microscopy, Direct Fluorescent Antibody, Immunohistochemistry, and Silver Stains** | | | | | | | | |
| Buffet et al [14] | - Prospective  - **Skin biopsy** from 12 patients with **secondary syphilis** diagnosis based on serology and clinical presentation and 24 control patients with dermatologic lesions  - **DFM** performed on lesions  - **NAAT *tpp47*** (260bp) and human *alb* internal control on immediately frozen fresh tissue  - NAAT amplicon detected by Southern Blot for 25 bp region and sequenced  - **IHC on FFPE using avidin-biotin peroxidase complex technique with polyclonal Abs (BioCare**)  - Semi-quantitative eval in epidermal, superficial and deep dermal layers | | - IHC positive in 11/12 (91.7%) biopsies from syphilis patients; spirochetes predominantly in epidermis and superficial to medium dermis  - NAAT positive in 9/12 (75%) syphilis patients  - NAAT Limit of detection determined 1 ng DNA  - DFM positive in 7/12 (58%) patients  - 1 patient DFA positive/IHC and NAAT negative  -All negative controls were negative by all assays  - *alb* detected in all NAAT assays | | - Limit of detection studies not described well  -Well characterized secondary syphilis samples (clinical and serology)  - Low number of samples  - DFM not well described | | - IHC higher sensitivity than *tpp47* PCR  - Higher NAAT sensitivity than other studies – due to fresh snap frozen (-80C) tissue?  -Recommend both IHC and NAAT for secondary syphilis diagnosis  - *T. pallidum* localized to epidermis/upper dermis | |
| Romanowski et al [9] | - Single site, STD clinic, 1984-1985 enrollment Edmonton, Canada  - **Anogenital lesion exudate** collected from 128 patients and evaluated by **DFM** and **DFA testing using monoclonal H9-1 Ab to 47-48kDa *Tp* protein**  - Diagnosis of syphilis determined by:  - DFM positive, or  - New serology positive, or  - 4X increase in RPR | | - 66 patients with syphilis based on indicated criteria  - 52/66 (78.8%) DFM positive  - 48/66 (72.7%) DFA positive with H9-1 mAb  - 48/52 (92%) sensitivity of H9-1 mAb compared to DFM  - 62 patients without syphilis; dark-field microscopy and H9-1 mAb were negative in all 62 exudate samples | | -Large number of dark-field positive samples to compare to  - Well defined methods for assessment of DFA and dark-field slides  - Only one sample from each patient evaluated – does multi-sampling improve sensitivity? | | - High correlation of mAb H9-1 DFA positivity with DFM positive from anogenital lesion exudate | |
| Lee et al [11] | -**Retrospective** identification of 30 patients with confirmed syphilis (clinical and serologic)  - 37 **FFPE archived tissue biopsies** (5 primary, 31 secondary, 1 gastric mucosa)  - Biopsies tested by:  1) **Avidin-biotin-peroxidase complex** (ABC)  2) **Indirect Immunoperoxidase** (IIP)  3) **FTA-ABS Complement** (FAC)  - **DFM** performed in 3 primary and 14 secondary lesions  - Results of staining methods compared to clinical diagnosis | | - Compared to clinical diagnosis, ABC method most sensitive (35/37; 95%), followed by IIP (33/37; 89%), FAC (26/37; 70%) and Dark-field (12/17; 71%)  - Statically significant difference between ABC and FAC, DFM for secondary syphilis lesions | | - Included method-specific negative controls (omitting primary Ab or replacement with normal serum)  - Detailed methods provided  - Not all patients had dark-field performed (N=17) | | - ABC more sensitive due to multiple biotin molecules associated with secondary Ab and strong biotin-avidin affinity (vs. other conjugated Ab techniques)  - Immunoperoxidase methods in general more sensitive than DFM or FAC | |
| Hoang et al [18] | -**Retrospective** identification of 17 patients with secondary syphilis diagnosis confirmed by serology and histologic examination; 19 biopsy from 17 patients  - Biopsy stained with **avidin-biotin peroxidase complex (ABC)** technique (BioCare Medical *Tp* Ab) and **Steiner stain**  - 14 control biopsies presenting similar to secondary syphilis also evaluated | | - 12/17 (71%) positive by ABC and 7/17 (41%) positive by Steiner stain  - All controls negative by ABC; unclear if also negative by Steiner stain  - Statistically significant higher sensitivity of ABC method shown vs. Steiner (p=0.084)  - High level of melanin granule staining by Steiner stain | | - Limited number of positive samples  - Secondary syphilis cases only  - Controls included!  -Statistical analysis performed!  - Detailed description of histologic appearance of lesions and location of treponemes | | - ABC method more sensitive than Steiner Silver stain | |
| Daniels et al [12] | - Single site, **prospective**  - Study A: Compared **DFM vs. DFA** in 350 lesion exudates  - Study B: Compared DFA from lesion exudate vs. FTA-ABS in serum from 95 patients  - Syphilis diagnosed using the following criteria:  - Case history  - Clinical manifestations  - Serologic test results  - DFM results | | - 89.1% (312/350) agreement b/w DFM and DFA in Study A  - DFA sensitivity and specificity from both Study A and B was 85.9% (115/134) and 100% (311/311), respectively  - DFM sensitivity and specificity from Study A was 73.8% (87/118) and 97.4% (226/232), respectively  -7 samples from clinically diagnosed primary syphilis patients were DFM and DFA negative; 24 samples were DFM negative/DFA positive | | - Large number of samples evaluated  - Definition/criteria for diagnosis of syphilis (serology results, etc.) not well defined  - No statistics | | - DFA on acetone fixed lesion exudate is a sensitive method for direct detection of syphilis  - Lower sensitivity of DFM for detection of spirochetes in lesion exudate compared to DFA | |
| Hook et al [10] | - Hybridomas created to make mAb specific to Tp.  - **H9-1 mAb** specific to *T. pallidum* and *T. pertenue*, but not other human commensal treponemes  **- C2-1 mAb** also used, reacts with all spirochetes (cont.)  - 30 patients with syphilis based on DFM and serologic criteria and 31 patients without syphilis enrolled  - Lesion exudate collected from all for **DFM** and **DFA with H9-1 mAb**.  - Serum collected for FTA-ABS and VDRL | | - H9-1 mAb DFA was positive in 30/30 patients with syphilis and negativenegative in 31/31 patients without syphilis  - DFM was positive in 29/30 patients with syphilis and 2/31 patients without syphilis. Commensal spirochetes confirmed in the 2 patients using the C2-1 mAb | | - Detailed description of methods and criteria used for syphilis diagnosis  - Use of C2-1 mAb for spriochetes to confirm commensals | | - H9-1 mAb has high sensitivity and specificity for detection of T. pallidum from acetone fixed lesion exudate. | |
| Rawstron et al [21] | -17 **cases of fetal demise due to congenital syphilis (CS)** in RPR+/FTA-ABS+ mothers and **CDC criteria** identified  - Original tissue from autopsies restained with **Dieterle** and **indirect** **IFA using rabbit α-*Tp* sera (S. Lukehart)**  - Additional 116 sections from multiple organs from 16 fetuses with CS stained with Dieterle and IFA | | - Of original specimens, Dieterle was positive in 7/17 (41%) and IFA positive in 15/17 (88%)  - Of 116 tissue sections from 16 fetuses with CS, IFA positive in 63/116 (54%) and Dieterle positive in 43/116 (37%); IFA significantly more sensitivity than Dieterle (p<0.01)  - Tissue most common to have treponemes was lung and umbilical cord | | - Statistics performed  - Large number of tissue sections from multiple organs in 16 cases of CS  - Negative controls used from stillbirth fetuses in RPR negative mothers  - All stains read blindly  - Detailed information regarding organ sections from stillbirths was lacking | | - IFA more sensitive than Diterle stain in tissue sections from stillbirth fetuses with CS  - Most common sites of treponemes in lung and umbilical cord | |
| **2. Nucleic Acid Amplification Tests** | | | | | | | | |
| Lukehart et al [52] | - **Prospective**, single site  - Enrolled patients with serologic evidence of syphilis without prior treatment for syphilis  - **Sera and CSF** tested by VDRL and FTA-ABS  - 7 P syph  - 33 S syph  - 3 EL syph  - 15 LL syph  - **CSF** tested by **RIT** and compared to clinical stage | | - RIT positive in 12/58 CSF  - Positive in 2/7 primary and 10/33 secondary syph; negative in all latent cases  - Tp isolation by RIT higher in patients with ≥2 CSF abnormalities (protein, leukocytes, VDRL+)  -Neurologic signs present in only 6/33 patients with SS  -7/10 SS patients positive by RIT, were re-tested by RIT post-t’ment  - 3/3 patients with 2-3 penG doses were RIT neg  - 3/4 patients with 1 penG dose were RIT positive | | - Detailed evaluation of RIT performance in comparison to serologic findings, clinical presentation and CSF findings | | - Seminal study on detection of Tp in CSF using RIT  - Indicates dissemination of Tp into CSF even in asymptomatic individuals with primary or secondary infection  - Impact of treatment on RIT sensitivity | |
| Hay et al [53] | -Single site, **prospective**  - ***Tp tmpA* (45kDa) and *4D* (19kDa) NAAT** on **CSF** with confirmation by S. blot  - *Tp* distinguished from *T. pertenue* at 1 bp in *4D* gene, but this bp not in amplified product  - CSF positive for *Tp* only if both NAAT reactions positive  - 3 grps of patients tested:  - A (N=30: No history of syphilis/HIV  - B (N=19): Syphilis positive patients (TPHA and FTA positive on serum, with or without positive VDRL), concern for neurosyphilis  - C (N=28): HIV+ patients undergoing LP for quesrion of CNS disease  - **Neurosyphilis diagnosis by symptoms and positive VDRL in CSF**  - Possible neurosyphilis diagnosis if abnormal CSF, VDRL neg, but FTA/TPHA CSF pos | | - 1/30 (3.3%) patients in Grp A without History of *Tp* were NAAT pos  - Grp B: 10/19 (53%) NAAT positive in CSF, including both patients with VDRL positive CSF, 1/2 patients with possible neurosyphilis, and 7/14 patients with latent syphilis (late and tertiary syph)  - Grp C: 14/28 patients with CNS disease of these 14, 7 were *Tp* NAAT positive and all 14 patients without CNS disease were NAAT neg  - Overall:  -59% sens, 97% spec, 91% PPV, 83% NPV in patients without prior syphilis tment  - 38% sens, 87% spec, 87% PPV, 62% NPV in HIV+ patients with history of syph  - CSF stored in liquid N_2_ until testing  - Limit of detection: 65 org/0.5 mL | | - Retrospectively determined that primer pairs used for testing were not 100% specific. 2/3 false positives were negative using nested NAAT for 4D gene  - No other comparator assays (*e.g*., RIT) | | - NAAT on CSF may help confirm diagnosis of neurosyphilis in suspicious cases, though a negative result does not r/o infection | |
| Castro et al [35] | - Single site, enrollment of 124 patients with syphilis reactive blood tests  -**Neurosyphilis (NS) Diagnosis based on European guidelines (French et al. 2009)**  - + CSF TPHA and/or FTA and increased CSF WBC (>5/mm^3^) or RPR/VDRL+  - **Syphilis staging based on 2014 European guidelines (Janier et. al)**  - 100 CSF from patients syphilis seronegative included as controls  - ***tpp47* (Orle)** and ***polA* (Liu)** **NAATs** performed on all **CSFs**; products visualized by EtBr on gel | | - All 100 CSF controls were negative by both NAATs  - 37/124 (29.8%) CSFs positive by *tpp47* PCR  - Of 33 with NS criteria, 25 (75.6%) were *tpp47*+  - Of 91 without NS criteria, 12 (13%) were *tpp47*+  - 30/124 (24.2%) CSFs positive by *polA* PCR  - Of 33 with NS criteria, 23 (69.7%) were *polA*+  - Of 91 without NS criteria, 7 (7.7%) were *polA*+  -Majority of NAAT discrepancies in patients with latent syphilis | | - Specific ‘syphilis reactive bld test’ results not defined for enrollment criteria  - Ref. stand. used included clinical criteria and serology  - No further analysis of discrepant samples | | - *tpp47* NAAT higher sensitivity vs. *polA* NAAT higher spec in CSF  - Overall sens/spec for NS NAATs:  -*tpp47*: 75.6%/87%  -*polA*: 69.7%/92.3%  - NAAT on CSF sens/spec in patients with NS symptoms meeting European criteria for NS  - Utility/sensitivity of NAAT in asymptomatic NS cases is questionable | |
| Orle et al [28] | -Single site, USA STD clinic, collection of **genital lesion exudate** (N=298)  - Multiplex **NAAT for *tpp47****,* HSV and *H. ducreyi*  - **Colorimetric detection with biotinylated-olgiont probes**  -**DFM** and **RPR/VDRL** used as comparators  - **Individual *Tp* NAAT to basic memb protein gene used for discrepant analysis** | | -65/298 samples DFM pos, of which 58 also positive by M-NAAT for *Tp*.  -17 samples M-NAAT +/DFM -, of which 15 also positive by individual *Tp* PCR. 2 sampes were M-NAAT false+  - 91% (66/73) sensitivity of M-NAAT to DF/individual Tp NAAT  - 88% sensitivity of DFM compared to M-PCR/individual Tp PCR  - 99% and 100% spec for M-NAAT and DF, respectively | | - Comparison to RPR/VDRL not included since no treponemal serology used to confirm testing  - Combination of reference standards used to evaluate M-PCR  - Extensive analytical specificity panel performed  - 7 M-NAAT false negatives may have been due to sampling error due to multiple swabs of same lesion  - Limit of detection studies performed (10 org) | | - M-NAAT and individual NAAT for Tp from genital lesions more sensitive than dark field microscopy | |
| Zoechling et al [20] | -**Retrospective** selection of **13 FFPE** from **secondary (N=6) and tertiary (N=7)** cases of syphilis confirmed clinically, by histopathalogy findings and serology  - 5 negative control biopsies, 6 non-Tp spirochete and 1 Tp culture slide controls included  - ***tpp47*** NAAT from 10um FFPE cuts used; beta-actin internal amp control used | | - All 13 were Warthin-Starry negative  - 4/6 secondary syphilis biopsy were PCR+  -1/7 tertiary syphilis biopsy were PCR+; positive samples was from **gumma** | | -Blades and gloves changed b/w each cut and reaction  -No comparison to DFM, IHC or other | | - Superior sensitivity of *tpp47* NAAT vs. traditional means (silver stains) in biopsy material  - Higher sensitivity in secondary vs. tertiary syphilis | |
| Marfin et al [38] | - Single site, **outbreak investigation**  - WB collected from 32 patients with either signs of syphilis or who were a sexual partner of someone with syphilis  - Samples originally collected for molecular typing via *tpr* and *apr* genes  - **WB** tested for ***Tp polA*** and amplicons visualized by EtBr stain of gel | | - 7 patients with incubating, 7 patients with primary, 1 with secondary, 13 with latent and 4 with non-syphilis lesions  - 13/28 (46%) WB samples from patients with syphilis were *polA*+; all 4 WB samples from non-syphilis patients were negative by PCR  - *polA*+ in  - 8/13 latent cases  - 3/7 incubating cases  - 1/7 and 1/1 PS and SS  - No correlation b/w RPR titer and MHA-TP reactivity and NAAT positivity | | - No confirmation of NAAT amplicons using S. blot  - Small number of patients  - No RIT | | - *Tp polA* NAAT on WB may be used to detect syphilis in patients with latent infection  - NAAT may be useful regardless of serologic level of reactivity  -Spirochetemia occurs throughout course of infection and may be detected by PCR | |
| Palmer et al [29] | - **Prospective** enrollment of patients with ulcers at STD clinics  - Swabs (dry or in viral transpo media) tested by ***tpp47* NAAT** (Orle 1996) and viewed by EtBr  - 98 patients evaluated, 100 specimens tested  - **Results compared to clinical diagnosis/staging and serologic results**  - **DFM** performed on 34/100 (34%) samples | | - 18/19 (95%) of patients with PS were PCR+; 2/10 (20%) were DFM+  - 8/10 (80%) of patients with SS were PCR+; 0/3 were DFM+  -23/23 patients with HSV or past, treated syphilis were PCR-; 9/9 were DFM-  - 48 patients with Diagnosis of ‘Not syphilis’, 1/48 PCR+ and 1/12 DFM+  - Compared to clinical/serologic Diagnosis, NAAT had sens/spec/PPV/NPV for PS and SS of 95%/99%/95%/99% and 80%/99%/89%/97%, respectively | | - Comparison of NAAT to clinical diagnosis (presentation and serologic findings), guidelines not cited  - Limited number of ulcer samples tested by DFM; none tested by DFA for *Tp*  - Amplicon visualization using EtBr only  - No RIT | | - *tpp47* NAAT helpful in distinguishing HSV vs Tp ulcers in primary disease | |
| Castro et al [39] | - Single site in Lisbon  - 69 patients with **indeter. latent syphilis** (no symptoms, RPR/MHA-TP/FTA-Abs+)  -18 patients **treated for syphilis,** tested 6m post-treatment  - Collected:  - **WB, Plasma, Serum, Ear lobe scrapings**  - **Orle 1996 *tpp47* NAAT** and **Lui 2001 *polA* NAAT** used; EtBr gel detection of amplicons  - *tpp47* and *polA* NAATs also used as multiplex | | -NAAT results for each target for the 69 patients meeting **CDC criteria for latent syphilis** are as follows:  ***tpp47* PCR+**  - WB+: 27/69 (39.1%)  - Plasma+: 31/69 (44.9%)  - Serum+: 18/69 (26.1%)  - Ear lobe+: 16/28 (57.1%)  ***polA PCR+***  *-* WB+: 19/69 (27.5%)  - Plasma+: 25/69 (36.2%)  - Serum+: 14/69 (20.3%)  - Ear lobe+: 15/28 (53.8%)  ***Tpp4 and/or polA M-*PCR+**  - WB+: 26/69 (37.7%)  - Plasma+: 29/69 (42%)  - Serum+: 19/69 (27.5%)  - Ear lobe+: 16/28 (57.1%)  -*tpp47* was the only NAAT positive in 3 patients  - No sample *polA* or M-NAAT positive only; both NAATs were positive in 2 patients negative by *tpp47*  *-tpp47* PCR+ in 39.1% (92/235 samples) of patients with latent syph; M-NAAT positive in 38.3% (90/235 samples) | | - *Tp* Nicols strain positive cont and syphilis seronegative patient DNA for negative controls used  - No RIT | | - *Tp* not detected by NAAT post complete treatment of syphilis  - Highest sensitivity for latent syphilis NAAT achieved in earlobe scrapings>plasma>WB>serum  -*tpp47* NAAT had higher sensitivity vs. *polA* PCR | |
| Martin et al [26] | - Two-site, STD clinic **prospective** enrollment of patients with suspected syph; 87 specimens from 68 patients  - **Patients staged based on clinical and serologic findings according to Canadian STI guidelines**  - **WB, serum CSF and swab from ulcers/lesions**  - **NAAT for *bmp, polA* and *tpp47*; std NAAT for *tpp47***  - Beta-glob amp control included  - EtBr visualization of amplicons | | - All NAATs gave concordant results in all tested samples  -Sensitivity depends on specimen source  - 41/68 patients diagnosis with syphilis (53 specimens from 41 patients)  - 19 primary; 9 secondary; 10 latent; 3 congenital  - 14/41 (34%) patients and 19/53 (36%) of samples PCR+  - 9/19 primary syphilis PCR+  - 9/12 (60%) swabs with Beta-glob amplification were PCR+  - 0/9 WB+  - 4/9 secondary syphilis PCR+  - 4/9 (44%) WB+  - 1/1 swab+  - 1/3 WB from congenital case was PCR+  - 0/10 latent cases was PCR+ in WB, swab or CSF  - All 27 nonsyphilis cases were PCR- | | - No differentiation b/w performance of each NAAT rxn  -Small number of cases  - No RIT, DFM | | - NAAT on swabs from primary syphilis lesions highest sensitivity; WB not sensitivity source for primary syphilis cases  - WB better NAAT source for secondary syphilis, though still <50% sens  -NAAT in WB/swab/CSF not sense for latent syphilis | |
| Gayet-Ageron et al [30] | -Multi-site, **prospective** enrollment of patients with **suspected syphilis and matched case-controls (!!)**  - Comparison of ***tpp47* RT-NAAT (Taqman)** performance in **WB, urine, swabs** and/or **CSF** for HIV+ and HIV- patients  -**Syphilis cases staged based on clinical and serologic data/CDC guidelines** | | - 74 patients enrolled/126 specimens collected  - 38 patients/47 specimens *tpp47* RT-PCR+ and discussed  - Overall sensitivity in:  - Primary 17/26 (65%)  - Secondary 21/40 (53%)  - Latent 0/8  -No difference in sensitivity in HIV+ vs. HIV- patients, though HIV+ patients with secondary syphilis had lower Ct values (high spirochetemia)  - Primary syphilis sensitivity by source:  - Lesion swab 8/10 (80%)  - WB: 5/18 (28%)  - Serum: 6/11 (55%)  - Urine 2/7 (29%)  - Secondary syphilis sensitivity by source:  - Lesion swab 1/5 (20%)  - WB: 11/31 (36%)  - Serum: 7/15 (47%)  - Plasma: 2/2 (100%)  - Urine 4/9 (44%)  - CSF: 3/6 (50%)  - High specificity of assay – all controls negative. | | - No RIT, DFM, etc.  - First to evaluate urine  - No control of antibiotic use prior to collection  - Not all specimens received from all patients | | - Urine sensitivity 29-44%  - Highest sensitivity from lesion swabs for primary syphilis (80%)  - Highest sensitivity from serum, WB, plasma for secondary syphilis (36-100%)  - NAAT off any source not useful in latent syphilis  - No difference in NAAT performance in HIV- vs. HIV+ | |
| Behrhof et al [17] | - **Retrospective** study using 39 archived **FFPE** collected between 1954-2006 in Germany  - **36 skin biopsy** from patients with secondary syphilis (33 confirmed by serology and 3 not confirmed)  - **Nested (Tp1; 228 bp) and semi-nested (Tp2; 125 bp) NAAT for DNA polymerase I** performed on all samples.  - Gel and direct seq. Internal bcl-1 control tested.  - Samples tested by **Dieterle silver stain and IHC** **using rabbit polyclonal antibody (Biocare)** | | - Tp1 and Tp2 nested NAATs positive in 7 and 14 of the 36 FFPE, respectively  - 23/36 FFPE had positive internal bcl-1 control by PCR  - 11/23 positive by Tp2 and 6/23 positive by Tp1 NAATs  - 17/35 FFPE positive by IHC (positive defined as presence of >3 spirochetes)  -9/35 FFPE positive by Dieterle | | - Internal controls for NAAT and positive/negative controls for staining used  - Nested PCR, amplicons run on gel and direct sequencing for interpretation = risk for sample contamination  - 13 samples failed to amplify internal control on NAAT and were unusable  - No statistics performed b/w sources/methods | | - NAAT more sensitive than Dieterle staining  - In cases where DNA quality is good, NAAT has similar sensitivity to IHC | |
| Cruz et al [19] | - Multi-site enrollment at **STI clinics** in Colombia of patients with **suspected secondary syphilis** (57 enrolled)  - **Cases defined using serologic (RPR>=1:4 and FTA-ABS+) and clinical criteria (charc. lesions)**  - **WB and biopsy** collected and tested by **NAAT for *Tp polA* (TaqMan)**; inhib control (human RNase P gene) tested for samples negative for *Tp*  - **Biopsy also stained with Warthin-Starry** | | - Skin biopsy from 11 patients with secondary syph; Warthin-Starry positive in 1 (9%)  - *Tp polA* NAAT Limit of detection was 15-150 spirochetes/ml; Limit of detection 1 log higher in samples stored at 4C for 26hrs vs. RT for 1hr  -WB collected in 26 patients; 12/26 (46%) *Tp polA*+  -Copies/mL ranged from 195-1954; not correlated with RPR titer  -No inhibition control in NAAT negative samples  -*Tp polA*+ in 8/12 (66%) biopsies tested | | - No RIT  - Not all enrolled patients tested by NAAT in WB or biopsied  -Study focused on epi of SS in Colombia  - Small numbers; no statistics performed comparing sources | | - *Tp polA* RT-NAAT higher sensitivity in skin biopsy from patients with SS (66%) vs WB (46%) | |
| Grange et al [13] | - Multi-site (2 STD clinics in Paris) enrolling patients with suspected syphilis and 35 healthy volunteers  - **Diagnosis of syphilis and staging based on CDC criteria**  -**DFM** on lesions from primary and secondary  -**NAAT for *tpp47*** on **lesion exudate** collected by sterile swab and all blood fractions (**WB, PBMCs, serum, plasma**) | | -Limit of detection: 20 org/mL  - 65 patients with primary syph  - 49 (75%) DFM+ vs. 52 (80%) NAAT+; p=0.011  - 44 patients with secondary syph  - 31 (70%) DFM+ vs. 38 (86%) NAAT+; p=0.339  - Sn/Sp/YI/PV/NV/LR^+^/LR^-^ for DFM in 1^st^ and 2^nd^ syph:  72/88/0.61/94/57/6.3/0.31  - Sn/Sp/YI/PV/NV/LR^+^/LR^-^ for NAAT in 1^st^ and 2^nd^ syph:  82/95/0.78/98/68/18/0.18  - NAAT concordance with diagnosis was 82.6%  - DFM/NAAT concordance rate 77%; kappa 0.53  - NAAT in blood fractions:  - 12-38% sensitivity range  - 24% overall sensitivity; 97% overall spec, 39.9% concordance btw NAAT and diagnosis  - PBMCs highest sensitivity in primary syphilis 32% (23/72)  - WB highest sensitivity in secondary syphilis 38% (26/69) | | - Oral lesions not excluded from dark field microscopy  - Amplicons visualized by EtBr on gel  - No RIT | | - NAAT may be used as replacement/in addition to DFM for assessment of lesions suspicious for syph  - NAAT on blood has low sensitivity; serum is worst source overall | |
| Yang et al [32] | - Single site, prospective enrolling 240 patients (267 episodes) with **any stage of syphilis defined by CDC STD T’ment guidelines, RPR >/=1:4 and TPPA+**  - **Oral swabs** on all patients, with or without oral ulcers; swabs of **genital lesions, plasma** from patients with secondary or early latent syphilis, **CSF** from patients with neurosyph  - Samples collected b4 Abiopsy  - ***polA* NAAT (Liu 2001** assay) on all samples  - **Typing using *arp, tpr, tp0548***  - Macrolide resistance by 23S PCR | | - All patients were male, 242/267 (91%) of tests in HIV+  - 22/267 (8.2%) episodes had oral syphilitic ulcers  - 38 cases of primary syphilis, 17 had oral ulcers, *polA*+ in 13/38 (34%) of samples and 13/17 oral ulcer cases  -*polA*+ in 113/267 (42%) oral swabs  - 64.5% of oral swabs from patients with secondary syphilis were *polA*+, regardless of lesion presence/absence  -*polA*+ in 28% and 40% of oral swabs from patients with early or late latent syphilis, resp.  -Detection of *polA*+ in oral swabs assoc with younger age, secondary syphilis, oral lesion, RPR >1:32  - Higher yield rates for *polA, arp, tpr, tp0548* in patients with oral ulcers vs. those without  - 45/113 oral swabs amplified all 3 serotyping genes  -*Tp* serotype matched in 31/33 patients where DNA recovered from multiple sites | | - Staging based on clinical criteria; lesions may be overlooked/subtle  -Biased towards HIV+ males | | - Rate of *polA* detection from oral swabs is high, even among patients without lesions, particularly in secondary syph | |
| Heymans et al[27] | -Single site, **prospective** STI Clinic, Amsterdam  - Enrolled patients with suspected primary or secondary syph  -**DFM** on all anogenital lesions  - **RT-NAAT (TaqMan) for *polA*** on all **lesion swabs** or **skin scraping** (secondary syph)  - 3 Reference Stds for NAAT utility in primary syph:  1. NAAT vs. DFM  2. NAAT vs. clinic-based diagnosis which incorporates DFM, prior syphilis episodes, serology results  3. NAAT vs. clinic-based diagnosis without DFM  - Criteria for secondary syph  - ‘Skin manifestation possibly related to secondary syphilis (macular/popular rash, condyloma latum, roseolas, mucous patches, alopecia)’ and RPR >/=1:8 | | -93/716 (13%) and 34/133 (26%) suspected primary and secondary *polA*+  - *polA* vs. DFM sens/spec:  87% (47/54) and 94% (616/662); kappa 0.61  - *polA* vs. primary ref. std. #2 sens/spec:  73% (83/114) and 98% (592/602), kappa 0.769, PPV 89%, NPV 95%  - *polA* vs. primary ref. std. #3 sens/spec:  74% (76/102) and 97% (597/614); kappa 0.745  - *polA* vs. secondary syphilis sens/spec:  43% (33/77) and 98% (55/56); kappa 0.372 | | - Unclear if DFM from swab (probably swab) vs/ directly from lesion  - Limitations of clinical diagnosis, no mention of serologic results; no use of std diagnostic guidelines  - Limited discussion/explination of NAAT discordant samples | | - Fair agreement b/w *polA* and DFM  - *polA* NAAT better correlation with disease when compared to clinical/serologic assessment for primary syphilis  - Low NAAT sensitivity for secondary syphilis | |
| Gayet-Ageron et al [31] | - **Multi-center, prospective**, observational (2011-2013)  - Enrolled patients with genital, anal, oral ulcers suggestive of syphilis  - **3 Definitions of syphilis**:  - + DFM  - + serology (CDC algorithm)  - enhanced: clinical findings, serology +, DFM – indicative of FN DFM  - Ulcer material collected in standardized process (not defined)  - **NAAT for *tpp47* by Taqman performed on all samples**  - 273 patients; 53 of 226 were HIV+ | | - DFM done for 170 patients  - 32 DFM+ of which 30 were PCR+ (93.8% sens)  - 138 DFM= of which 13 were PCR+ (90.6% spec)  - NAAT compared to Enhanced def of syphilis (n=170):  - 16 symptomatic, serology +, DFM= patients were NAAT positive (87.5% sens/99.2% spec).  -Reliability of DFM strongly assoc. with routine performance | | - Strengths: prosptective, multi-site, patients included would likely most benefit from syphilis PCR, standardized NAAT performed at one location  - Limitation: lack of std. protocol for serologic assessment, lack of routine DFM performance at some sites; no differentiation bwith HIV+ or HIV-  - Results similar to Grange et. al and Heymans et. al | | - Show higher sensitivity of NAAT vs. DFM in well characterized patients with primary syphilis | |
| Hollier et al [15] | - **Prospective**, single site enrollment of **VDRL/MHA-TP positive pregnant** women at least at 24 wks gestation  - **AF** collected for **RIT**, **DFM** and **NAAT for *tpp47*** using Grimprel et al 1991 assay  - Funipuncture performed for VDRL and IgM by WB  - Maternal syphilis staged by clinical criteria (CDC 1985 guidelines) | | - 24 women met criteria; 6 primary, 12 secondary, 6 early latent  - 16/24 (67%) fetuses determined to have CS based on sonography and testing; therefore 67% transmission rate  -3/6 in primary cases  - 8/12 in secondary case  - 5/6 in early latent cases  - Amniotic Fluid:  - 5/21 (24%) DFM positive  - 12/20 (60%) RIT positive  - 10/20 (50%)NAAT positive  - 5 positive by all 3 assays  - 14 positive by ≥ 1 assay  - Compared to RIT, DFM had a sensitivity of 41% (5/12) and NAAT a sensitivity of 82% (9/11) | | - RIT used and DFM and NAAT evaluated | | - RIT has highest sensitivity for detection of *Tp,* followed by NAAT on AF | |
| Nathan et al [42] | - Single-site; **11 pregnant women** b/w 14-19 wks gestation with untreated early syphilis enrolled  - **AF** tested by **RIT** and **NAAT for *tpp47*** (658 bp) with S. Blot confirmation  - IgM for *Tp* and VDRL evaluated in all neonates | | - 4/11 (36%) AF samples positive by RIT  - 3/11 (27%) AF samples positive by PCR  - All NAAT positive were RIT pos; 1 RIT positive was NAAT neg  - 9/11 (82%) neonates were negative for IgM to *Tp*  - All neonates had VDRLs equal to or less than mom’s at delivery | | - Few studies have evaluated utility of AF testing during early gestation for diagnosis of syphilis  - Evaluated direct detection and serologic testing  - DNA hybridization used for confirmation of PCR | | - RIT has higher sensitivity for CS in AF fluid compared to PCR, though both low. | |
| Michelow et al [36] | - Single site study; enrolled 148 **newborns whose mothers were diagnosed with syphilis during pregnancy**  - **CSF** tested for *Tp* by **RIT** and compared to clinical findings, radiography, and other laboratory testing:  - ***Tpp47* NAAT on serum, plasma, blood, CSF**  - **RIT on blood, serum**  - IgM blotting on serum, CSF | | - 27/148 (18%) blood/serum RIT positive  - 19/148 (13%) CSFs positive by RIT  - CSF collected in 76 babies prior to Abiopsy  - RIT positive in 17/76 (22%)  - NAAT positive in 11/76 (14%)  - NAAT v. RIT sens: 65%  - Of 17 babies with confirmed CNS CS:  - RIT+ in 13/14 sera/blood  - PCR+ in 16/17 sera/blood  - Blood NAAT good predictor of CNS infection (sensitivity 16/17 and specificity 53/59) using RIT in CSF as reference method  - 4 infants with normal CSF findings and VDRL negative were RIT positive in CSF | | - No information on maternal diagnosis during pregnancy | | - Administration of Abiopsy prior to CSF collection associated with lower RIT sensitivity  - NAAT on blood best predictor of CSF infection (vs. serologic testing) when RIT in CSF used as reference method. | |
| Sanchez et al [37] | | - Single site; enrolled 19 **mother-infant pairs**. Mothers with untreated early syphilis, HIV neg  - 7 symptomatic and 12 asymptomatic infants at birth  - ***tpp47* NAAT** and S. blot confirmation  - **RIT** performed on all infant **CSF** and some serum  -VDRL and IgM blot on all infant CSF/serum | | - 7 symptomatic infants:  - sera positive by RIT in 3/3 samples; NAAT positive in 6/7 sera, including the 3 RIT pos  - RIT positive in 6/7 CSF; NAAT positive in 5/7  -12 asympto infants:  - RIT positive in 2/2 sera tested; NAAT positive in 2/12; 1 sera RIT and NAAT pos  - RIT positive in 1/12 CSF; NAAT negativenegative in all 12 CSF; VDRL NR in 11/12 CSF  - Overal NAAT vs. RIT in CSF (n=19):  - 71% (5/7) sensitivity  - 100% (12/12) specificity  - kappa: 0.76  - Overall NAAT vs. RIT in CSF and sera (n=29 samples):  - 75% (9/12) sensitivity  -100% (17/17) specificity  - kappa: 0.79  - 5 infants with RIT/NAAT positive CSF pre treatment were CSF neg. 2-8 months post treatment | | - Used RIT as gold standard  - No mother-infant controls enrolled  -Post treatment RIT/NAAT data not shown | | - Tp NAAT on CSF of symptomatic infants is helpful adjunct for detection of congenital syphilis  - NAAT on CSF of asymptomatic infants with congenital syphilis has limited sensitivity, similar to RIT |
| Grimprel et al [16] | - Single site  - **AF** from 11 **pregnant** with untreated syphilis  - **Sera & CSF** from 20 **neonates** with probable or suspected congenital syphilis  - Pregnant mothers Diagnosis using conventional criteria; all neonates evaluated at birth  - Control patients from preg. women and neonates without syph  - **Goal**: a) evaluate different methods to minimize NAAT inhibition and b) compare NAAT to RIT on diff sources  - **DFM** on AF  - **RIT** performed on **AF, CSF and sera**  - 4 different DNA preparation methods for NAAT evaluated  - NAAT for ***Tpp47*** gene with Southern blot confirmation | | - 9/11 (82%) AF samples from untreated pregnant women with syphilis positive by NAAT using low-spin extraction method; 6 AFs **DFM** positive. 9 had RIT on AF and 7 were RIT Positive  - RIT and NAAT performed on CSF of 18 neonates and positive in 5 and 3, respectively  - RIT performed in sera from 5 neonates and positive in all 5; NAAT performed in all 20 sera and positive in 9. All but 1 RIT positive sera was NAAT pos  - Compared to RIT, NAAT sensitivity in AF, neonate CSF and neonate sera was 100% (7/7), 60% (3/5) and 67% (4/6), respectively; overall sensitivity of 78 (14/18)%. Specificity was 100%  - NAAT in sera 2-7m post treatmentment negative by NAAT in nenoates previously RIT or NAAT positive in sera | | | - RIT performed and used as gold standard  - Negative patient controls incorporated  - Sensitivity difference may be due to different volumes used for RIT (0.5-2 mL) vs NAAT (10-100ul)  -Specimen storage conditions were not optimal following collection and DNA may have degraded (not immediately placed in -70 | | - NAAT may be useful adjunct to Diagnosis and management of congenital syphilis pre-treatment |

| **Supplemental Table 3. Table of Evidence summarizing molecular epidemiology testing options for *T. pallidum*.** | | | | |
| --- | --- | --- | --- | --- |
| **Citation** | **Description of study type, design, population, and setting** | **Reported findings, quantitative results related to key question** | **Overall quality; strengths/weaknesses or limitations** | **Relevance to the key question and/or overall importance** |
| Katz et al [46] | -Single site, San Francisco STD clinic, 2004-2007  -Swabs from 74 primary or secondary syphilis patients that were Dark-field positive  -Detection NAAT for *polA*  -Typing NAAT for *arp/tpr* genes with RFLP  -Additional testing for *arp* negative samples using alternate primer set and touchdown PCR  -Additional typing by determining number of G tandem repeats in *rpsA* gene | -70 primary syphilis and 4 secondary syphilis patients  -69/74 samples could be fully typed; 66 primary and 3 secondary syphilis cases  -Using 3 gene (*arp, tpr, rpsA*) typing scheme, 8 subtypes were identified, 14d9 being most frequent (52/69) | - Subtyping based on *rpsA* not described previously  - Limited number of samples  - No controls for *rpsA* | - Additional subtyping ability using *rpsA* gene to better characterize outbreak types |
| Marra et al [45] | -Multi-site  - 72 *Tp* isolates from bld, CSF, exudate (Seattle)  -11 historical *Tp* isolates  -16 bld samples (Seattle)  -74 primary or secondary lesion swabs from Madagascar, San Francisco, Baltimore, China, Ireland  - Evaluated strain types b/w blood and CSF in 84 patients with neurosyphilis  - Evaluated typing using *arp/tpr* NAAT and RFLP **AND**  RFLP analysis of *tprC* (*tp0017), tprD (tp0131)*, sequencing of *tp0548,* and presence of 51bp insertion between *tp0126/tp0127* | -51bp insertion and RFLP analysis of *tprC* did not improve discrimination vs. Pillay 1998  -Sequencing of bases 131-215 of *tp048* divided 14d subtype into 3 groups and subtypes 12a and 14a each into two groups.  -*tp048*  sequencing and *arp/tpr* typing = strain type  -173 samples were typed into 14 *arp/tpr* subtypes and 24 *arp/tpr/tp048* strain types  -Confirmed stability of typing method following multiple passages of Nichols strain in rabbits  -84 samples from patients with neurosyphilis: able to show that 14d subtype able to be further strain typed and 14d/f more neuroinvasive compared to 6 other detected strain types | -Related enhanced strain typing to clinical disease (neurosyphilis) and showed that sequencing of *tp048* allowed for discrimination within the CDC 14d subtype  -Showed stability of *tp048* sequence over serial rabbit passages  -Additional studies needed to confirm relevance of *tp048* sequencing  -Unclear what syphilis detection NAAT was performed  -Statistics performed | -Additional typing using *tp048* sequencing may be used to further strain type isolates for epidemiologic purposes |
| Pillay et al [43] | - Lab type strains and clinical samples collected from GUD patients in the US, Madagascar and S. Africa  - Clinical samples confirmed for *T. pallidum* by M-PCR  -Specificity of *arp/tpr* NAATs determined by M-NAAT negative GUD samples  -GUD swabs in Roche NAAT transport medium, stored at -70C until processing | - Identified two genes, *arp* and *tpr* subfamily II gene family (*tprE, tprG, tprJ*) with intra-strain variability enabling a subtyping method  - Hybrid typing system using the number of 60 bp repeats of *arp* and RFLP analysis of *tpr* gene using *MseI* enzyme.  -16 subtypes identified among 46 isolates in study  - Discriminatory value of typing system determined by Simpson’s Index of Diversity: D value 0.868 | -First typing method described  - Limited number of clinical samples, yet identified 16 subtypes  -Found no change in typing pattern of Nichols strain after passage in rabbit for 1.5 years  -Define NAAT methods for *arp, tpr* and RFLP analysis  - Used negative controls | -Original study describing *arp/tpr* RFLP based gene subtyping for *T. pallidum* |
| Florindo et al [44] | - STI clinic in Lisbon, Portugal  -416 patients with suspected early syphilis enrolled b/w 2004-2007  - Primary and secondary skin lesion exudate and blood collected  - Samples screened by *bmp* NAAT and confirmed by *polA* NAAT (Lui 2001)  -*arp/tpr* subtyping using modified Pillay 2002 protocol | -86/416 (42 lesion exudates and 44 blood samples) patients positive by syphilis *bmp* and *polA* NAATs  - Successful genotype from 27/42 (64%) lesion exudates and 15/44 (34%) blood samples; 42/86 (49%) overall successful subtyping  - Detected strain types 14a, 14d, 14f | - Used patients with suspected syphilis, not confirmed serologically; dark field not performed  - Compared skin lesions (assume exudate ?) vs. blood as sources  -No discussion of handling/collection/storage of specimens prior to testing | - Subtyping from blood was less sensitive compared to skin biopsy  - Lower sensitivity in blood possibly due to inhibitory substances or lower spirochetemia |
| Castro et al [41] | - STI clinic in Lisbon, Portugal  - 82 patients (212 specimens) enrolled b/w 2003-2005 with clinical symptoms and serology positive for syphilis (all three stages)  - Specimens collected include plasma, blood, ear lobe scrapings, lesion exudate  - Samples screened by *polA* NAAT (Liu 2001) and typed (Pillay 1998) by *arp/tpr* NAATs/RFLP | **-** 90 (42%) specimens positive by *polA*; highest sensitivity in lesion exudate (15/16, 94%) and ear lobe scraping (21/32, 65%), lowest in blood (22/82, 27%)  - 62/90 (69%) subtyped; highest sensitivity in lesion exudate (13/15, 87%) and ear lobe scraping (16/21, 76%), lowest in blood (13/22, 59%)  - Detected strain types 10a, 14a, 14c, 14f, 14g  - Samples processed and held at -4C for 1-2 days before processing | - Use of ear lobe scrapings for syphilis detection  - Ear lobe scrapings may be used in patients without other lesions  - Limited number of samples, not equal b/w specimen types | - Blood has lowest sensitivity for direct detection by *polA* NAAT and subtyping by *arp/tpr*  - Highest subtyping percentage from lesion exudate collected by swab, followed by ear lobe scraping using scalpel and swabbing blood using swab and placed in PBS |
| Molepo et al [48] | -1999-2000, 50 CSF specimens from patients with suspected neurosyphilis in Pretoria, S. Africa; single site  -CSF tested by VDRL, FTA-ABS; neurosyphilis confirmed if either positive  -*tpp47* gene for detection NAAT and subtyping by *arp/tpr* (Pillay 1998)  -RFLP *tpr* typing using *Tru91*, isoschizomer of *MseI*  -CSF stored -70 | -35/50 CSF were VDRL/FTA-ABS positive and 5/50 FTA-ABS positive only  -28/50 CSF were *tpp47* NAAT pos  -15/28 with sufficient DNA for typing; 13 fully typed and 2 partially typed  -4 strain types identified | - Limited number of positive samples  - 5 VDRL/FTA-ABS negative samples were NAAT positive…FPs vs. TPs? No discordant analysis | -VDRL positive CSFs have higher probability of containing sufficient DNA for typing  -One of two studies on typing from CSF |
| Martin et al [49] | -Single site, Shanghai STD clinic 2007-2008  -39 patients with symptoms of primary syphilis (GU)  -Syphilis Diagnosis confirmed by Dark-field and/or serology  -Serum (TRUST and TP-PA), whole blood and GU swabs collected  -10 swabs from non-syphilis GU collected for controls  -Swabs in saline and stored -70C  -Detection NAAT using *tpp47, bmp,* *polA*  -Control for NAAT inhibition by NAAT for beta-globulin in syphilis NAAT negativenegative samples  -Subtyping using *arp/tpr* (Pillay 1998) | - 39 patients with primary syphilis confirmed by dark-field (N=35) and serology (N=4)  -38/39 Tp NAAT positive for all 3 genes (!) from GU lesions; negative in all blood samples  - identified 5 subtypes, 14f most common | - Blood and GU lesions tested  - Confirmed detection of *T. pallidum* DNA using 3 genes in well characterized primary syphilis patients  - Negative and internal controls used | - Blood poor specimen source for syphilis detection PCR, including use of buffy coat or whole blood  - High sensitivity in GU lesions |
| Lu et al [40] | - Single site, enrollment of 3024 pregnant women in China  -41 RPR and TPPA positive from whom WB, plasma, ear lobe scrapings and mucosal lesion exudate (if present) were collected  - *tpp47* screening NAAT on all raw samples; positive samples typed using *arp, tpr* and *tp0548* | - 13/41 (32%) WB *tpp47*+  - 8/13 (61%) typed  - 17/41 (41%) plasma *tpp47*+  - 12/17 typed  - 25/39 (64%) earlobe scrapings *tpp47+*  - 21/25 typed  - 10/11 exudates *tpp47+*  - 10/10 typed  - Highest *tpp47* positivity in all sources from secondary syph | - Small number of patients in evaluated despite high enrollment | - DNA detection/typing from lesion exudate and earlobe scrapings provides highest sensitivity |
| Xiao et al [47] | - Cross-sectional, multi-site study in China dermatovenereologic clinics  - WB collected from patients with secondary or latent syphilis confirmed by Chinese guidelines and serology  - 2253 WB samples from 603 primary and 1650 secondary syphilis cases  - WB screened for *Tp* DNA with *polA, tpp47, bmp, tp0319* NAATs  - Screen pos. typed with *arp, tpr, tp0548* NAATs | -455/2253 (20.2%) of samples positive by at least 1 screening PCR; 306/2253 (13.6%) positive by all 4 NAATs  - Indiv. positive rates: 18.8% *polA*, 18.4% *tpp47*, 17.5% *bmp*, 14.2%  *tp0319* - 272/603 (45.1%) secondary syphilis and 183/1650 (11.1%) latent syphilis patients positive by ≥1 screening PCR  - *tpr, arp, tp0548* amplified from 181/455 (39.8%) of samples | - Other stages of syphilis not evaluated  - Limited success at typing  - Early vs. late latent syphilis not differentiated | - WB from secondary and latent syphilis cases associated with limited overall success rate for *Tp*  typing |
